# Differential neural dynamics underling pragmatic and semantic affordance processing in macaque ventral premotor cortex

**DOI:** 10.1038/s41598-019-48216-y

**Published:** 2019-08-12

**Authors:** Monica Maranesi, Stefania Bruni, Alessandro Livi, Francesco Donnarumma, Giovanni Pezzulo, Luca Bonini

**Affiliations:** 1 Department of Medicine and Surgery, University of Parma, via Volturno 39, 43125 Parma, Italy; 2 Center for Neural Science, New York University, New York, NY United States of America; 3 Department of Neuroscience, Washington University, St. Louis, Missouri USA; 4 Institute of Cognitive Sciences and Technologies, National Research Council, via S. Martino della Battaglia 44, 00185 Rome, Italy

**Keywords:** Decision, Premotor cortex

## Abstract

Premotor neurons play a fundamental role in transforming physical properties of observed objects, such as size and shape, into motor plans for grasping them, hence contributing to “pragmatic” affordance processing. Premotor neurons can also contribute to “semantic” affordance processing, as they can discharge differently even to pragmatically identical objects depending on their behavioural relevance for the observer (i.e. edible or inedible objects). Here, we compared the response of monkey ventral premotor area F5 neurons tested during pragmatic (PT) or semantic (ST) visuomotor tasks. Object presentation responses in ST showed shorter latency and lower object selectivity than in PT. Furthermore, we found a difference between a transient representation of semantic affordances and a sustained representation of pragmatic affordances at both the single neuron and population level. Indeed, responses in ST returned to baseline within 0.5 s whereas in PT they showed the typical sustained visual-to-motor activity during Go trials. In contrast, during No-go trials, the time course of pragmatic and semantic information processing was similar. These findings suggest that premotor cortex generates different dynamics depending on pragmatic and semantic information provided by the context in which the to-be-grasped object is presented.

## Introduction

Grasping an object requires to select the most appropriate hand posture (e.g. precision or power grip) to interact with it^1–4^ but also to plan the whole sequence of movements that will lead to successfully accomplish the agent’s behavioural goal (e.g. eating or placing the object, see^5–7^).

The neuronal circuits underlying visuomotor transformation of objects features into motor plans for grasping them have been extensively investigated. Existing data indicate that the intraparietal area AIP contains neurons responding to the visual presentation of specific objects as well as during the execution of the grip type required for grasping them^8^. Neurons with similar properties have been also found in the parietal area V6A^9^. Areas AIP and V6A are strongly and reciprocally connected with the premotor areas F5 and F2, respectively^10–12^, where visuomotor neurons with object-type specificity have been found^2,13–15^. Most recently, the mesial premotor area F6^16,17^ has been shown to constitute an additional node of the “cortical grasping network” - a set of richly interconnected parieto-frontal pathways underlying the sensorimotor transformation of object’s physical properties in the most appropriate hand posture to interact with it^18^. These circuits highlight the relevance of the “pragmatic” representation of observed objects^19^.

In the last decade, neurophysiological studies also highlighted a role of reciprocally interconnected parietal and frontal regions^20,21^ in organizing and planning goal-directed manual actions^5–7^. These studies showed that neurons responding during a grasping act performed with a given type of grip (e.g. precision grip) can exhibit markedly different activations depending on the final goal of the action (i.e. eating or placing). In fact, “what to do” with an object largely depends on its behavioural significance for the subject (e.g., palatability or repugnance), and both parietal and premotor neurons appear to be sensitive to this “semantic” information. A recent study^22^ also showed that area F5 grasping neurons respond differently to objects with the same size and shape (i.e. same “pragmatic affordances”) depending on whether they were edible or inedible (i.e. different “semantic affordance”). These findings suggest that the premotor cortex, in addition to pragmatic affordances, can also process semantic affordances, which might be relevant to make decision and planning forthcoming motor acts belonging to a specific goal-directed action^23,24^.

Yet, the possible differences between the processing of pragmatic and semantic affordances have not been systematically investigated. Thus, in this study we comparatively investigated the time course of F5 neuronal activity collected during different previous studies focused on pragmatic^15^ or semantic^22^ object affordances both at the single-neuron and population level. We found that the processing of pragmatic and semantic affordances has distinct neuronal signatures, which are coherent at both levels of analysis. Specifically, we report *sustained* activation during the processing of pragmatic information in contrast to *phasic* activation during the processing of semantic information, at both single-cell and population levels. This evidence suggests that premotor neuronal population processes visually presented objects flexibly depending on task context and demands. Furthermore, neural space analysis evidenced that the dynamics underlying semantic processing of observed objects resemble those emerging during object presentation in the No-go condition of both tasks. The phasic activation of premotor neuronal population during the processing of semantic information may therefore index a rapid suppression of premotor system’s dynamical activity.

## Results

**Figure 1:**
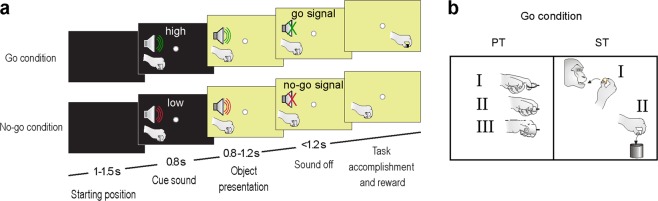
Temporal phases of PT and ST tasks and schematic representation of the Go condition. (**a**) The tasks were constituted by two main conditions: the Go condition (Grasping in the light - above) and the No-go Condition (Object fixation - below). The two conditions had the same temporal sequence, but in the No-go condition the low-frequency tone (300 Hz sine wave) instructed the monkey to remain still for 1.2 s, while in the Go condition the high-frequency tone (1200 Hz sine wave) indicated to the monkey to reach and grasp the target. (**b**) Schematic representation of the Go condition in both tasks: in the PT, monkey had to perform the correct grip (I - hook grip; II - side grip; III - whole hand prehension) as conveyed by the presented object; in the ST, monkey had to grasp-to-eat (I) or grasp-to-place (II) the target.

Four monkeys were trained to perform a visuomotor Go/No-go task in which an auditory cue initially instructed the animal to either grasp or refrain from grasping a subsequently presented object (Fig. 1a). The sequence of task events was the same for all monkeys. Two of them (M1 and M2) were presented with three different non-edible objects of different size and shape (pragmatic task, PT, Fig. 1b). The other two (M3 and M4) were presented with two different objects, an edible and a non-edible one, both with the same size, shape and weight, but different colour (semantic task, ST, Fig. 1b). Previous studies provide a more extensive description of the details of PT^15^ and ST^22^.

**Figure 2:**
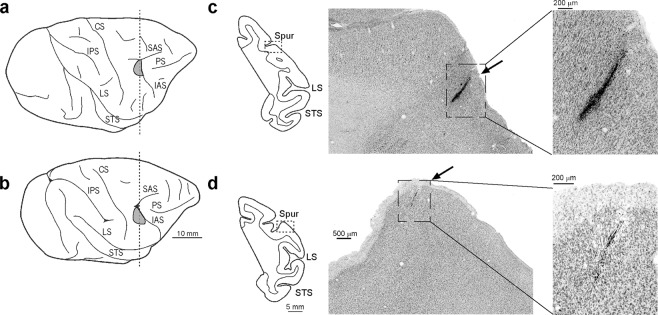
Lateral views of the hemispheres of M1 (**a**) and M4 (**b**). (**c**,**d**) Coronal sections taken at the level of the recorded regions, as indicated by the dashed line in (**a**,**b**). Low-power photomicrographs show higher magnification views of a Nissl-stained coronal section. Arrows indicate tracks of probe penetrations where visuomotor grasping neurons were recorded. For the sake of comparison, the data of M4 (left hemisphere) are shown as a right hemisphere. CS, central sulcus; IAS, inferior arcuate sulcus; IPS, intraparietal sulcus; LS, lateral sulcus; PS, principal sulcus; SAS, superior arcuate sulcus; Spur of the arcuate sulcus; STS, superior temporal sulcus.

Single neuron activity was recorded from the hand field of the medial part of area F5 in all four monkeys. Figure 2 shows the lateral view of the brain of two monkeys, one studied with the PT (Fig. 2a), the other with the ST (Fig. 2b). The reconstruction of two probes’ traces in Nissl-stained coronal sections (Fig. 2c,d) provides evidence of the close anatomical correspondence between the locations of the investigated regions in the two animals.

### Single neuron and population response

We recorded a set of 225 visuomotor neurons discharging during both object presentation and grasping execution, separately described in previous studies with other purposes^15,22^: 106 were recorded with the PT, and 119 with the ST. The proportion of neurons with an object-selective visual response did not significantly differ between the two tasks (45/106 in PT and 37/119 in ST, χ^2^ = 3.12, p = 0.077), despite the different number of target objects (three in the PT and two in the ST).

**Figure 3:**
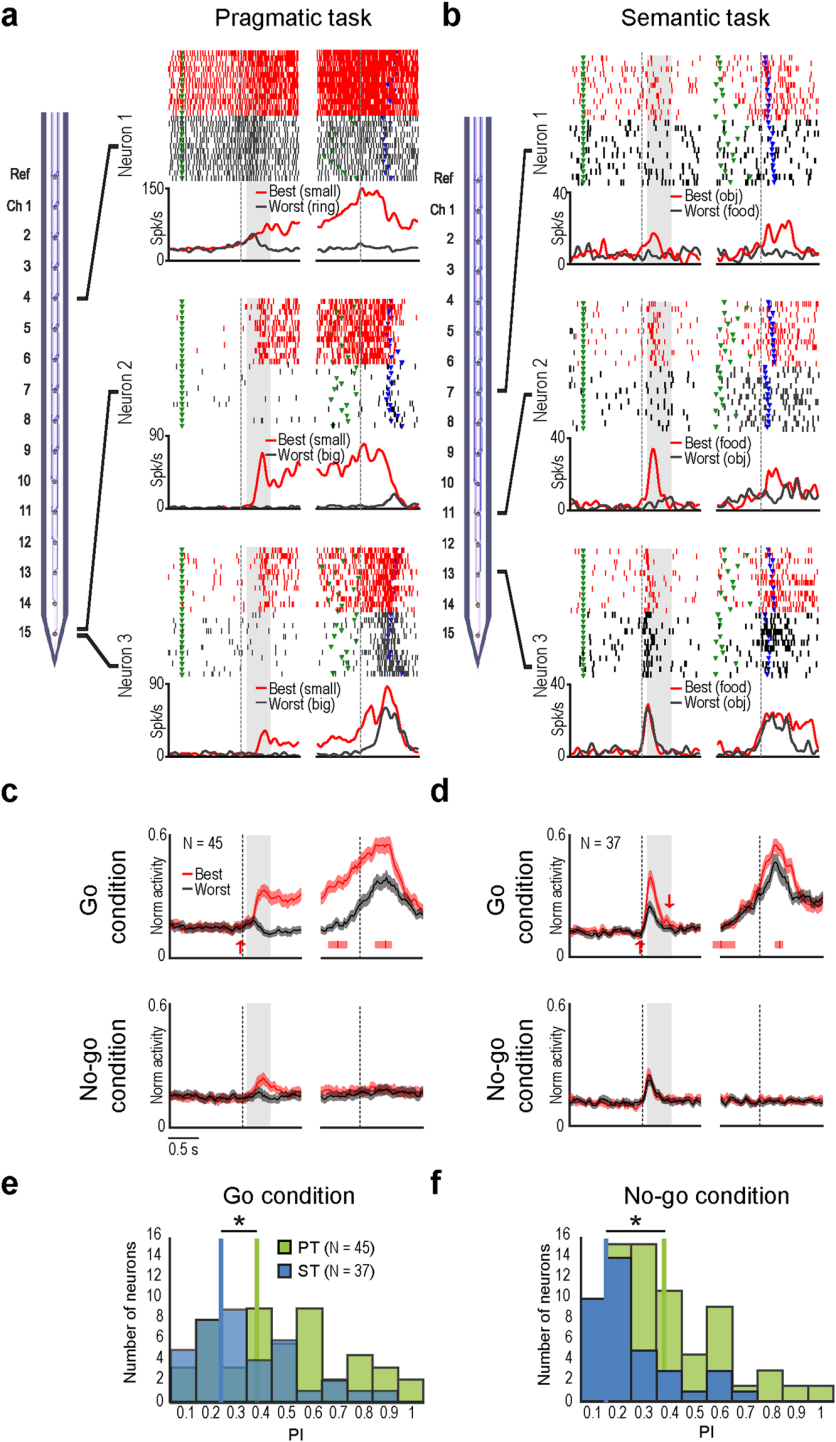
(**a**,**b**) Schematic drawings of linear multielectrode probes and examples of single units simultaneously recorded in PT (**a**) and ST (**b**). For each neuron, the gap in the histogram and rastergram is used to indicate that the activity on the left side has been aligned on object presentation (light onset - first vertical dashed line in the left panel), whereas the activity on the right side is aligned on detachment of the monkey’s hand from the starting position (reaching onset - second vertical dashed line in the right panel) of the same trial. The gray shaded areas represent the time windows used for statistical analysis of neuronal response. (**c**,**d**) Time course and intensity of the firing rate of the neuronal populations showing selectivity for the type of object during PT (at the left) and ST (at the right) relative to the best (red) and worst (grey) object, in the Go (above) and No-go (below) conditions. The activity is aligned to object presentation (light onset - vertical line on the left), and reaching onset in the Go condition (detachment of the monkey’s hand from the starting position - vertical line on the right), as well as to the sound off in the No-go condition (vertical line on the right). The red and grey shaded regions around each curve represent 1 SE. Upward and downward arrows indicate the time when the population response to the best object became - or ceased to be - significantly different from baseline, respectively (sliding t-test in 200 ms bins performed in steps of 20 ms, p < 0.05, corrected, for at least 3 consecutive bins from −80 to + 700 relative to object presentation). (**e**,**f**) Frequency distribution of preference indexes (PIs) for visually presented objects during Go (**e**) and No-go (**f**) conditions of both tasks (ST - blue; PT - green). *t-tests p < 0.05.

Figure 3a shows examples of three visuomotor neurons simultaneously recorded with the PT. Neuron 1 showed the strongest discharge during the visual presentation of the small cone, and the weakest discharge to the presentation of the ring. Similarly, Neuron 2 and 3 exhibited the same visuomotor preference for the small cone, but different discharge intensity during trials with the other objects. Figure 3b shows examples of three visuomotor neurons simultaneously recorded with the ST, each exhibiting different visuomotor preference for the edible (Neuron 4) or inedible (Neuron 5) object, or no preference (Neuron 6). It is worth to note that all the example neurons recorded with the PT show sustained activation after object presentation (Fig. 3a), in line with several previous studies on area F5 visuomotor neurons^2,14,15,25^, whereas none of those recorded with the ST (Fig. 3b) exhibit such an activation pattern. The time course of population activity during both Go and No-go conditions (Fig. 3c,d) further demonstrates that object coding in ST is more strictly related to the behavioural context than in PT. Indeed, whereas in the PT the difference between population coding of the best and worst object defined on the basis of single neuron response during the Go condition remains significant also during No-go condition (t = 5.5, p < 0.001), this does not occur in the ST (t = 0.9, p = 0.39). Furthermore, the distribution of Preference Indexes (PI, see Methods) for visually presented objects in the two tasks evidences that object selectivity is greater in the PT than in the ST during both Go (t = 2.8, p = 0.007) and No-go (t = 2.9, p = 0.0046) conditions (Fig. 3e,f). Importantly, even if the PIs are calculated considering all the three objects used in the PT, the differences between the two tasks remain the same for both Go (t = 2.2, p = 0.0293) and No-go (t = 3.04, p = 0.0031) conditions.

**Figure 4:**
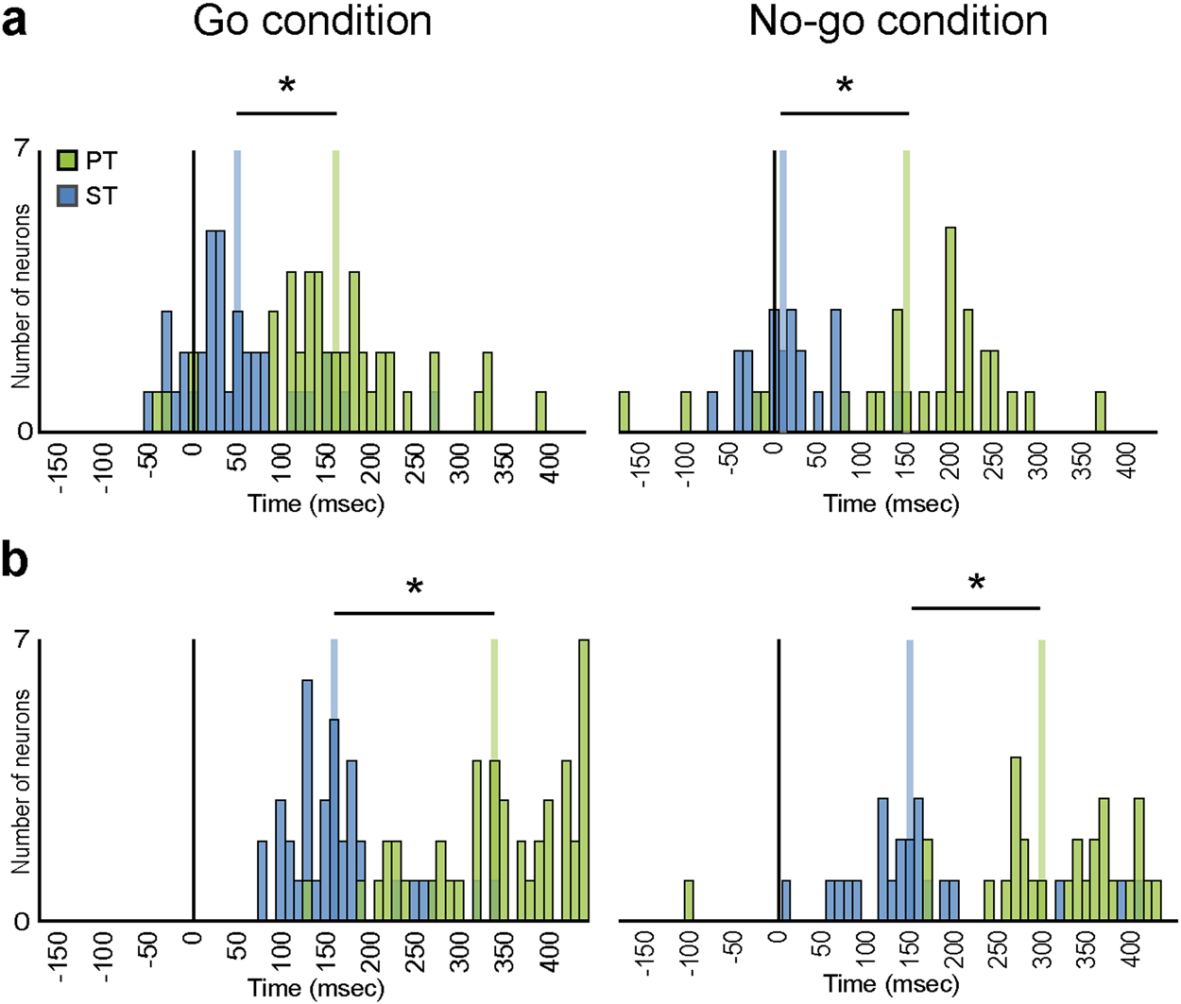
Frequency distribution of discharge onset (**a**) and peak of activity timing (**b**) of neurons with object-selective visual response in the Go condition calculated during Go (left) and No-go (right) conditions of both tasks (ST - blue; PT - green). In the No-go condition, only neurons with significant object presentation response have been included (22/37 in ST and 29/45 in PT). *Significant difference between the two distributions, t-tests p < 0.05.

To better understand the time course of pragmatic and semantic affordance processing, we next calculated the discharge onset and peak of activity time (see Methods) for each recorded neuron in both tasks (Fig. 4). We found that neurons recorded in the ST began to discharge earlier (mean ± standard deviation for onset: Go condition 48.6 ± 65.4 ms; No-go condition 17.7 ± 48.7 ms) and reached their peak of activity sooner (peak: Go condition 164.3 ± 60.6 ms; No-go condition 160.9 ± 98.1 ms) than those recorded during PT (mean onset: Go condition 160.7 ± 88.5 ms; No-go condition 162.8 ± 115.5 ms; peak: Go condition 344.4 ± 81.8 ms; No-go condition 311 ± 105.6 ms) (P < 0.001 for all comparisons). The discharge onset and peak activity time showed no significant difference (P > 0.05) between the two monkeys performing each task, thus suggesting that the results most likely reflect genuine differences between the tasks rather than inter-individual differences between animals. On these bases, it appears that pragmatic processing of an observed object entails a slow and sustained recruitment, in PMv, of the neural motor representation that can be used to grasp it, whereas the processing of semantic features rapidly and transiently modulates the same neurons with a less evident linkage to subsequent motor preparation and action execution processes.

### Neural space analysis

A recently proposed hypothesis maintains that some essential components of motor and premotor function can be better characterized by their population dynamics in a low-dimensional “neural space” than by the classical approaches based on representational tuning of single neuron and population data^26^, as those employed so far in this work. Thus, we adopted dimensionality reduction techniques (i.e. principal component analysis, PCA) to perform a so-called neural space analysis^27–31^ of premotor population dynamics. This analysis should allow us to better scrutinize the relationship between the neuronal signatures so far described and population dynamics in the temporal processing of pragmatic and semantic information about observed graspable objects. We found that, regardless of the task, the first three principal components explain a large and similar portion of variance of the neural data (73% for M1, 68% for M2, 70% for M3 and 72% for M4). Thus, both tasks appear to be equally relevant to probe the function of the investigated region, and the first three components of PCA convey significant information about population dynamics.

**Figure 5:**
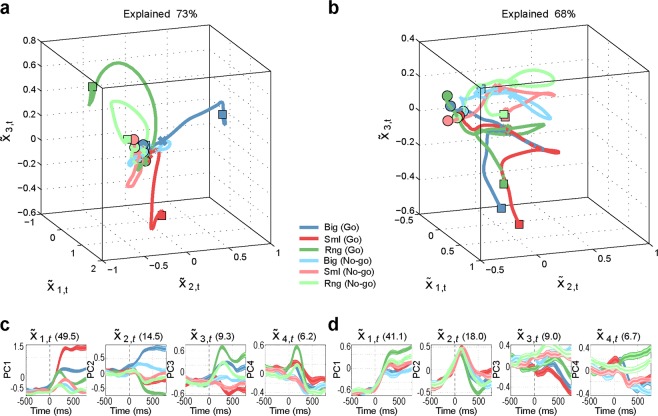
Neural space analysis, pragmatic task (PT). (**a**,**b**) Trajectories in the 3D “neural space” formed by the first three principal components of PCA, during the pragmatic task (PT) performed by M1 (**a**) and M2 (**b**). The neural trajectories span the interval [−500, 800], with zero being the target onset, and each bin representing the average of 200 ms. Circles mark the beginning of the neural trajectories, crosses mark target onset, and squares mark the end of the neural trajectories. (**c**,**d**) Trajectories in the 1D space of the first 4 components of PCA (one panel for each component) for M1 and M2, respectively. Mean (coloured line) and variance (coloured shades) are shown.

Figure 5 shows the results of neural space analysis carried out in monkeys M1 (Fig. 5a) and M2 (Fig. 5b) during PT (Supplementary Fig. S1 shows the results of the same analysis with aggregated data). The figures show six trajectories within the 3D neural space made of the first three principal components. Each trajectory corresponds to the mean population dynamics in each of the six conditions of PT. In both monkeys, the six trajectories start from a common point and then diverge during Go and No-go conditions (see Methods for a description of a quantification of the proximity of the neural trajectories over time in terms of “spread” in neural space. In this task, some differences emerged between the two monkeys. In M1, the trajectories associated with the various objects are separated, and reach distinct endpoints during Go condition, whereas during No-go condition they briefly diverge and then tend to collapse to the same point, close to the start, in the neural space (Fig. 5a). In M2, the trajectories in Go and No-go conditions from two distinct clusters that move away from the starting point (Fig. 5B).

Despite these differences between animals, in both M1 and M2 the trajectories in Go conditions tend to be more sustained as compared to the trajectories in No-go conditions (e.g. in the first two principal components of both monkeys). Furthermore, in both M1 and M2 the neural trajectories form two distinct clusters for Go and No-go conditions, suggesting a crucial role of neural population dynamic during PT in specifying whether to move (Go) or refrain from moving (No-go), depending on the context. Interestingly, in both M1 and M2 the clusters for Go condition are wider than the clusters for No-go condition (see Supplementary Fig. S2 for a quantification of the “spread” in neural space). This implies that in both M1 and (to a lesser extent) M2, the neural trajectories distinguish different objects more clearly during Go than No-go conditions.

**Figure 6:**
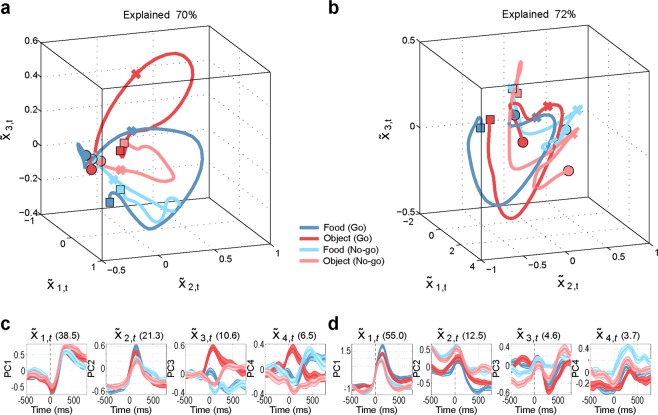
Neural space analysis, semantic task (ST). (**a**,**b**) Trajectories in the 3D “neural space” formed by the first three principal components of PCA, during the semantic task (ST) performed by M3 (**a**) and M4 (**b**). The neural trajectories span the interval [−500, 800], with zero being the target onset, and each bin representing the average of 200 ms. Circles mark the beginning of the neural trajectories, crosses mark target onset, and squares mark the end of the neural trajectories. (**c**,**d**) Trajectories in the 1D space of the first 4 components of PCA (one panel for each component) for M3 and M4, respectively. Mean (colored line) and variance (colored shades) are shown.

Figure 6 shows the results of neural space analysis in M3 (Fig. 6a) and M4 (Fig. 6b) during the ST (Supplementary Fig. S3 shows the results of the same analysis with aggregated data). Even in this task, some differences emerged between the two monkeys. Neural space analysis of M3 (Fig. 6a) shows that all the neural trajectories originate from a similar starting point in the 3D neural space, show tonic activation and then tend to return towards the same starting point. Interestingly, in this animal the semantic information (i.e. object identity, food versus non-food) is easier to discriminate as compared to the task condition (Go versus No-go). This is evident if one considers that the final points of the two object trajectories (red and orange squares) and the two food trajectories (blue and green squares) form clusters in neural space that are smaller than those formed by the two Go trajectories and the two No-go trajectories (see Supplementary Fig. S4). In contrast, in M4 (Fig. 6b), the trajectories of population activity in neural space originate from more heterogeneous spatial sectors, especially for No-go trajectories, and maintain a better separation between Go and No-go conditions than between the two objects over time (Supplementary Fig. S4).

Despite these differences, in both M3 and M4 the neural trajectories show more phasic activation, with smaller discriminability between conditions compared to PT (compare Figs S2a,b for PT with S2a,b for ST). Furthermore, the neural trajectories are quasi-circular and tend to return towards the starting point. Interestingly, unlike PT, this pattern is more homogeneous across both Go and No-go conditions (Supplementary Fig. S3).

Summing up, the distinction between Go and No-go conditions appears to be crucial in both PT and ST, although in ST the neural space analysis of one monkey (M3, see Fig. 6a) enhances the discrimination between semantic information on the target relative to the contextual instruction about moving or refraining from moving.

## Discussion

Seeing a graspable object within one’s own operative space triggers in the observers’ brain the neural representations of the potential actions they could direct to the object, that is, object’s affordances^18^. The Gibsonian concept of affordance assumes that a stable motor description of the object is linked with its physical properties^32^. In this vein, seeing a small object within one’s own reach should activate the same motor affordance (i.e. precision grip) regardless of the visual presentation context. In contrast, a recent study^22^ showed that area F5 neurons discharge differently when the monkey looks at small spherical objects having different semantic affordances (i.e., either edible or inedible). Interestingly, premotor neurons encode the identity of an object only when it is designated as a target for the subsequent behaviour. In other words, the “graspability” of the object is a prerequisite to evoke visual responses in various premotor areas, as demonstrated by recent studies where the same objects were made unreachable by the monkey because of their distance or by means of an interposed plastic barrier^15–17^. On top of pragmatic information, also semantic information can modulate object presentation responses. However, semantic modulation of object presentation responses seem to lack the typical sustained visual-to-motor pattern one would expect based on the findings reported in several previous studies^2,14,15,25,33^. In this work, we show that the neural processing of pragmatic and semantic affordances appears to be different at both single cell and population levels, suggesting that, in partial contrast with a strict Gibsonian view, the neural processing of object affordances can be flexible and strongly context-dependent^34,35^.

The results of the present study highlight, in particular, three main findings: 1) single neuron and population activity of area F5 encode differently pragmatic and semantic visual information about potential target objects; 2) pragmatic information about how to grasp a target induces sustained neural activation, at least during Go trials; 3) semantic information can be appreciated only in the context of action preparation or execution, because during the delay period single neuron and population activity suddenly returns to baseline - likewise during No-go trials.

The observed differences between single neuron and population dynamics during pragmatic and semantic visual information processing in F5 impose some preliminary methodological considerations. Indeed, it may be argued that they could depend on local anatomo-functional differences between the investigated sectors or by interindividual differences between the animals trained to perform PT and ST. However, we showed that the anatomical location and histological features of the investigated regions in all four animals were the same. Furthermore, differences in specific parameters of neural activity (e.g. discharge onset and peak activity time) were marked between tasks but not between animals performing the same task, strongly supporting the idea that there was no relevant difference between the pairs of monkeys performing each task. Hence, the observed temporal differences in the neural processing of pragmatic and semantic affordances can be reasonably attributed to distinct underlying visuomotor processes. In line with this idea, differences between sustained visual-to-motor activity and phasic visual and motor activity were observed with very similar versions of the PT^36^ and ST^37^, respectively, in ventrolateral prefrontal cortex. An alternative explanation is that the identical size and shape of the two objects in ST may have produced the lower visual selectivity relative to the PT. However, this phenomenon would be specific for the visual presentation response, because during reaching-grasping execution both parietal^5,6^ and premotor^6,21^ neurons can radically discriminate between two physically identical objects associated with a different behavioural meaning (i.e. edible vs inedible) and hence with a different final goal (i.e. eating or placing). Thus, the lower visual selectivity for semantic than pragmatic affordances is hardly accounted for by greater similarity between objects in the PT. A further criticism may be that the task design produced the observed differences between PT and ST. For example, the PT implies a degree of uncertainty in grip selection at the beginning of each trial, whereas this is not the case in the ST, where the monkey is always required to use a precision grip. Although it is known that the final goal of an action exerts an early influence on both neural^5,6^ and kinematic^38,39^ signature of intentional motor sequences, such as grasp-to-eat or grasp-to-place, in this latter case the major difference between the trials becomes more relevant when the object has been grasped and the task diverges. Future studies may directly explore this issue by testing the possible interaction between semantic and pragmatic affordance coding in single trials, randomizing within a unique factorial design the visual presentation of objects with various combinations of pragmatic (e.g. a small and a big object) and semantic (e.g. an edible or inedible) features.

A clear indication coming from the present study is that both single neuron activity and population dynamics are initially more deeply and steadily affected by pragmatic features, which are relevant for planning the next action stage, rather than by semantic information, which transiently modulate neuronal activity and then build up again later on during action unfolding. Indeed, neural space analyses of the PT revealed that, during Go trials, the neural population increasingly distinguishes the three objects, whose trajectories move towards three different subfields of the 3D space that potentially support optimal object-specific preparatory activity^30^. On the contrary, during No-go trials, the neural population tends to represent affordance information more transiently, rapidly losing object specificity (especially in M1). Interestingly, this latter loss of object specificity emerges in the neural space analyses of Go trials in the ST, suggesting they may be associated with premotor dynamics functionally similar to those of the No-go condition in the PT. A plausible explanation for this finding is that, in the ST, population dynamics are initially activated and then partially suppressed because monkeys plan and sequentially activate distinct motor chunks, likely corresponding to specific neural populations responsible for, e.g., hand and mouth control^39^, in line with several previous human studies^40–42^. Thus, the neural population controlling the initial motor action (e.g., hand grasping) may be suppressed by the activity of the neural population controlling the final action (e.g., mouth opening). This prediction may be tested in future studies by looking for cross-correlated and complementary activation/suppression of hand and mouth neuronal populations. This hypothesis fits well with the later and more spread visual response to object presentation observed in the PT, where it likely plays a role in the selection and preparation of the appropriate hand shape for reach-to-grasp. Instead, during the ST, motor preparation and hand shape selection are the same for both the food and the object, at least until the target is achieved. Indeed, only after this event the task diverges, and neuronal selectivity builds up again^21^.

Neural space analyses of the ST show a certain degree of variability between the two monkeys. Indeed, the trajectories are clustered based on semantic information in M3 and on task conditions (Go/No-go) in M4, indicating the existence of interindividual variability in population dynamics that may index different cognitive strategies. By looking at the time course of single principal components in M4, it is clear that the Go/No-go cue sound induces a separation of the neural trajectories associated with these two conditions already before object presentation, suggesting that M4, differently from the other animals, uses anticipatory strategies in action planning. This latter modulation may be most likely explained by top-down dynamics within hierarchical control schemes^38,43–46^ than by local neural mechanisms^47^, which may be more relevant during action unfolding. Further studies are needed to disentangle between these possibilities.

Despite the unavoidable interindividual differences, we also found some key results unaffected by them. First, only 3 components of PCA are sufficient to explain a large fraction of the variance of neural activity in all monkeys/tasks (ranging from 68 to 73%). Thus, prominent aspects of premotor cortex functioning can be reconstructed from the analysis of its low-dimensional dynamics. Second, the dynamics of the first principal components (i.e. those explaining more variance) differ between ST and PT, with the former appearing more phasic than the latter. This finding emerged also from PCA carried out on aggregated data from pairs of monkeys performing the PT (Supplemenetary Fig. S1) and the ST (Supplementary Fig. S3), indicating that the differences observed at the single neuron level can be reliably detected from low-dimensional population dynamics. Third, a difference emerges between Go and No-go conditions in PT and ST. No-go trials display less sustained activity than Go trials, but this difference is much more marked in the PT than in the ST, as also evidenced by the analysis on aggregated data (Figs S1 and S3). Indeed, during PT the trajectories in neural space for No-go trials (but not Go trials) tend to return back to the starting point. Rather, during PT, the trajectories for both Go and No-go trials show similar dynamics and tend to return back to the starting point. This evidence suggests that although both pragmatic and semantic information is relevant for action planning, pragmatic visual features of objects play a more important role than semantic ones in modulating premotor neuron activity.

## Methods

The experiments were carried out on four macaque monkeys (one *Macaca nemestrina* and three *Macaca mulatta*). Before recordings, the monkeys were habituated to sit in a primate chair and to interact with the experimenters. Then, they were trained to perform one of two versions of a sensory-cued Go/No-go task (Fig. 1) using the hand (left for M1 and M2; right for M3 and M4) contralateral to the hemisphere to be recorded (right for M1 and M2; left for M3 and M4). When the training was completed, a head fixation system and a plastic recording chamber were implanted under general anesthesia (ketamine hydrochloride, 5 mg/Kg intramuscular [i.m.] and medetomidine hydrochloride, 0.1 mg/Kg i.m., repeatedly administered during the surgery). Dexamethasone and prophylactic broad-spectrum antibiotics were administered pre- and postoperatively. Furthermore, analgesics were administered intra- and postoperatively. During all surgeries, hydration was maintained with continuous infusion of saline solution. A heating pad was used to maintain constant the temperature. The heart rate, blood pressure, respiratory depth and body temperature were continuously monitored. Upon recovery from anaesthesia the animals were returned to their home cages and closely monitored. All experimental protocols complied with the European law on the humane care and use of laboratory animals (directives 86/609/EEC, 2003/65/CE, and 2010/63/EU), they were authorized by the Italian Ministry of Health (D.M. 294/2012-C, 11/12/2012), and approved by the Veterinarian Animal Care and Use Committee of the University of Parma (Prot. 78/12 17/07/2012).

### Apparatus and behavioural paradigm

All monkeys were trained to perform a sensory-cued Go/No-go action sequence task. For two of them, the task included the visual presentation of three non-edible graspable objects, each of which afforded a specific type of grip but required the monkey to perform the same action, namely, grasping and pulling the object (Pragmatic affordance Task - PT - see^15^). For the other two monkeys, the task included the visual presentation of two graspable objects with the same size and shape, each of which afforded the same type of grip (precision grip) but required the monkey to perform a different action, namely, eating or placing the target in a container, depending on whether it was an edible or inedible one (Semantic affordance Task - ST - see^37^). Both tasks were implemented by means of the same apparatus described in the previous papers, and were characterized by the same sequence of instructional events (Fig. 1A). The monkeys were required to maintain fixation during the whole trial, and they were always automatically rewarded (with a drop of juice in PT and a food pellet in ST) after correct accomplishment of each trial.

#### Pragmatic affordance Task (PT)

Monkeys were presented with three different objects (a ring, a small cone, and a big cone), which afforded three of the most represented grip types used by macaques in their natural environment^48^, namely: hook grip (in which the index finger enters the ring), side grip (performed by opposing the thumb and the lateral surface of the index finger), and whole-hand prehension (obtained by opposing all the fingers to the palm). Objects were presented, one at a time during different trials, on the monkey’s body midline, within reaching distance from its hand starting position.

#### Semantic affordance Task (ST)

Monkeys were presented with two physically identical objects (small spheres, 6 mm of diameter and weighting 19 mg), which afforded the same type of grip (precision grip), but a different goal-directed action: eating, in case the target was a food pellet, and placing, when the target was a non-edible plastic sphere. As for the PT, objects were presented, one at a time during different trials, on the monkey’s body midline, within reaching distance from its hand starting position. The monkey could discriminate the behavioural value of the two objects only based on their colour: ochre for the pellet and white for the plastic sphere.

### Recording techniques

Neuronal recordings were performed by means of 16 channels silicon probes developed in the EU project NeuroProbes^49,50^ and distributed by ATLAS Neuroengineering (Belgium). Probes were inserted through the intact dura by means of a manually driven stereotaxic micromanipulator mounted on the recording chamber. All penetrations were performed perpendicularly to the cortical surface, with a penetration angle of approximately 40° relative to the sagittal plane. Previous studies provide more details on the devices and techniques employed to handle the probes^51^.

The recordings were carried out by means of an 8 channels AlphaLab system (AlphaOmega, Nazareth, Israel), and a 16 channels Omniplex system (Plexon, Dallas, Texas) (see^15,37^ for details). The wide band (300–7000 Hz) neuronal signal was amplified and sampled in parallel with the main behavioural events, and with the digital signals defining the task stages. All quantitative analyses of neuronal data were performed offline, as described in the subsequent sections.

### Recording of behavioural events and definition of epochs of interest

Distinct contact sensitive devices (Crist Instruments, Hagerstown, MD) were used to detect when the monkey touched with the hand the metal surface of the starting position, the objects (PT) or the floor of the groove hosting the target (ST) during grasping, and the metallic border of the plastic jar during placing of the object (ST). Each of these devices provided a TTL signal, which was used by LabView-based software to monitor the monkey performance.

Eye position was controlled by an eye tracking system composed by a 50 Hz infrared sensitive CCD video camera (Ganz, F11CH4) and two spots of infrared light. The analog signal related to horizontal and vertical eye position was fed to a computer equipped with dedicated software (Pupil), enabling calibration and basic processing of eye position signals. The eye position signals, together with the TTL events generated during task execution, were sent to the LabView-based software in order to monitor task unfolding, and to control the presentation of auditory and visual cues of the behavioural paradigm. Based on TTL and eye position signals, the software enabled the automatic interruption of the trial if the monkey broke fixation, made an incorrect movement or did not respect the temporal constraints of the behavioural paradigm. In all these cases no reward was delivered (for other details see^15,37^).

Since in this work we focus on neurons responsive to object presentation, we defined specific epochs of interest for statistical analysis based on the digital signals related to the main behavioural events: (1) baseline epoch, including the 500 ms before object presentation; (2) object presentation epoch, from 50 to 450 ms after stimulus onset (for details see Bonini *et al*., 2014a and Bruni *et al*., 2015).

### Data analyses and classification of the recorded neurons: single neuron activity

Single units were isolated using standard principal component and template matching techniques, provided by dedicate offline sorting software (Plexon), as previously described elsewhere^15^. After identification of single units that remained stable over the entire duration of the experiment, neurons discharge has been firstly analysed in order to identify motor neurons responding during grasping execution by means of specific repeated measures ANOVAs as described in the previous papers^15,37^. Briefly, we considered as “motor” all neurons discharging differently during hand-shaping and/or grasping-holding phase of at least one of the possible targets relative to baseline. Possible responses to object presentation relative to baseline were assessed in both Go and No-go conditions, by means of a 3 × 2 × 2 (PT) or 2 × 2 × 2 (ST) repeated-measures ANOVA (factors: object, condition, epoch), with a significance criterion of P < 0.01. Based on the results provided by these analyses, we could distinguish two main types of neurons: purely motor neurons, which responded during the execution of grasping actions but not to object presentation, and visuo-motor neurons, activated both during object presentation and grasping execution (purely visual neurons were virtually absent in the data set and have not been included in this work).

Each visuo-motor neuron firing rate, aligned on object presentation, was averaged on a trial-by-trial basis (*N* = 12) in a time range of −0.6 s and +0.58 s relative to the alignment point, using 200 ms bins slid forward in steps of 10 ms (*N* = 109 bins). The average firing rate in each bin during object presentation was then compared with that during baseline (paired-sample *t* test). The timestamp corresponding to the first of a series of (at least) seven consecutive bins with an uncorrected *p* value lower than 0.05 was taken as the discharge onset time. Moreover, the timestamp corresponding to the higher firing rate during the object presentation was taken as the peak activity.

### Data analyses: population level

Population analyses were performed on specific sets of neurons, classified on the basis of the results of single neuron analyses. Population vectors were computed by normalizing each neuron’s response across all the compared task conditions and epochs, averaged in a time window of 60 ms slit forward in steps of 20 ms. The same epochs employed for single unit data were also used for population analyses. To measure the object preference of specific neuronal subpopulation, we computed a Peference Index (PI) for each individual neuron according to Moody and Zipser^52^ with the following Equation:

$$PI=\,\frac{n-(\frac{\sum {r}_{i}}{{r}_{pref}})}{n-1}$$

where n is the number of objects used, r_i_ is the activity associated to each object, and r_pref_ is the activity associated with the preferred object. The index can range from 0 (lack of selectivity) to 1 (response to only one object). To check for possible differences in the distribution of PI values associated to each condition (Go/No-go) between the tasks, we performed paired-samples t test (P < 0.05).

### Data analyses: neural space analysis

For the “neural space” analysis of population dynamics^27–31^ we used a dimensionality reduction technique - Principal component analyses (PCA) - on a neuronal population of the four different monkeys: 65 neurons for M1, 41 neurons for M2, 45 for M3 and 107 for M4. Data were aggregated from 15 experimental sessions for M1, 11 for M2, 7 for M3, 11 for M4. We performed four separate PCA analyses, one for each monkey (M1 and M2 for PT; M3 and M4 for ST). The activity of each unit was collected from 90 trials in the case of pragmatic task (15 Big-Go, 15 Small-Go, 15 Ring-Go, 15 Big-No-go, 15 Small-No-go, 15 Ring-No-go) and 48 trials in the case of semantic task (12 Food-Go, 12 Object-Go, 12 Food-No-go, 12 Object No-go). Data were aligned to target onset, aggregated in bins of 200 ms and analysed in the interval [−500, 800], where zero is the target onset. We analysed 3D trajectories for the first 3 principal components (which explain a large part of the variance) and show the projections of the data on the first 4 principal components. Data analyses were conducted using standard Matlab routines for principal component analysis (PCA).

We also quantify the “spread” of the single-trial neural trajectories for each task and condition, that is, their distance in neural space (e.g., the 2D neural space formed by the first 2 principal components, see Figs S2 and S4). We operationalized the spread as the area (ellipsoidal confidence region) in neural space covered by (slices of) the single-trial trajectories, at different moments in time. A small/large spread indicates that - at a given moment - the single-trial trajectories of a specific condition (e.g., Go trials in PT, Big object) occupy a small/large subspace within the 12D neural space.

## Supplementary information

Supplementary Information
