# Author Correction: Differential neural dynamics underlying pragmatic and semantic affordance processing in macaque ventral premotor cortex

**DOI:** 10.1038/s41598-020-62216-3

**Published:** 2020-03-19

**Authors:** Monica Maranesi, Stefania Bruni, Alessandro Livi, Francesco Donnarumma, Giovanni Pezzulo, Luca Bonini

**Affiliations:** 1 Department of Medicine and Surgery, University of Parma, via Volturno 39, 43125 Parma, Italy; 2 Center for Neural Science, New York University, New York, NY United States of America; 3 Department of Neuroscience, Washington University, St. Louis, Missouri USA; 4 Institute of Cognitive Sciences and Technologies, National Research Council, via S. Martino della Battaglia 44, 00185 Rome, Italy

Correction to: *Scientific Reports* 10.1038/s41598-019-48216-y, published online 12 August 2019

The original version of this Article contained an error in the title of the paper, where the word “underlying” was incorrectly given as “underling”. This has now been corrected in the PDF and HTML versions of the Article and in the accompanying Supplementary Information file.

