# Supplementary material for: Differential neural dynamics underling pragmatic and semantic affordance processing in macaque ventral premotor cortex
Source: Sci Rep. 2019 Aug 12;9:11700. doi: 10.1038/s41598-019-48216-y (PMC6691108; doi:10.1038/s41598-019-48216-y)
Supplement: Supplementary file 1 — Supplementary Information [file 41598_2019_48216_MOESM1_ESM.pdf]

Supplementary Information

Differential neural dynamics underling pragmatic and semantic affordance processing in macaque ventral premotor cortex

Monica Maranesi[1]*, Stefania Bruni[1,2], Alessandro Livi[1,3], Francesco Donnarumma[4], Giovanni Pezzulo[4], Luca Bonini[1]

[1] Department of Medicine and Surgery, University of Parma, via Volturno 39, 43125 Parma, Italy.

[2] Center for Neural Science, New York University, New York, NY, United States of America.

[3] Department of Neuroscience, Washington University, St. Louis, Missouri, USA.

[4] Institute of Cognitive Sciences and Technologies, National Research Council, via S. Martino della Battaglia 44, 00185 Rome, Italy.

Monica Maranesi and Stefania Bruni contributed equally to this work.

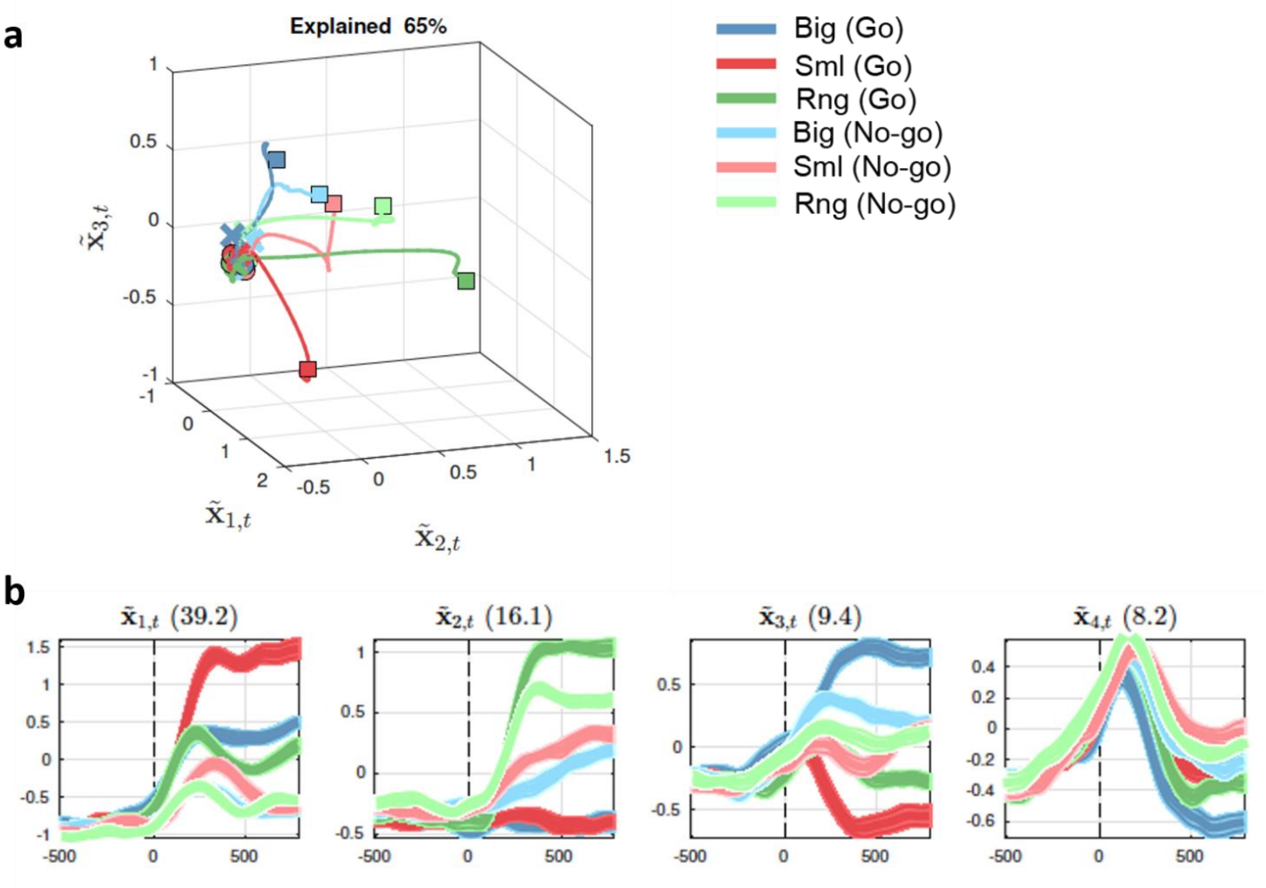

Figure S1. Neural space analysis with aggregated data from M1 and M2 - pragmatic task (PT). (**a**) Trajectories in the 3D "neural space" formed by the first three principal components of PCA, during the pragmatic task (PT). The neural trajectories span the interval [-500, 800], with zero being the target onset, and each bin representing the average of 200 ms. Circles mark the beginning of the neural trajectories, crosses mark target onset, and squares mark the end of the neural trajectories. (**b**): Trajectories in the 1D space of the first 4 components of PCA (one panel for each component). Mean (coloured line) and variance (coloured shades) are shown.

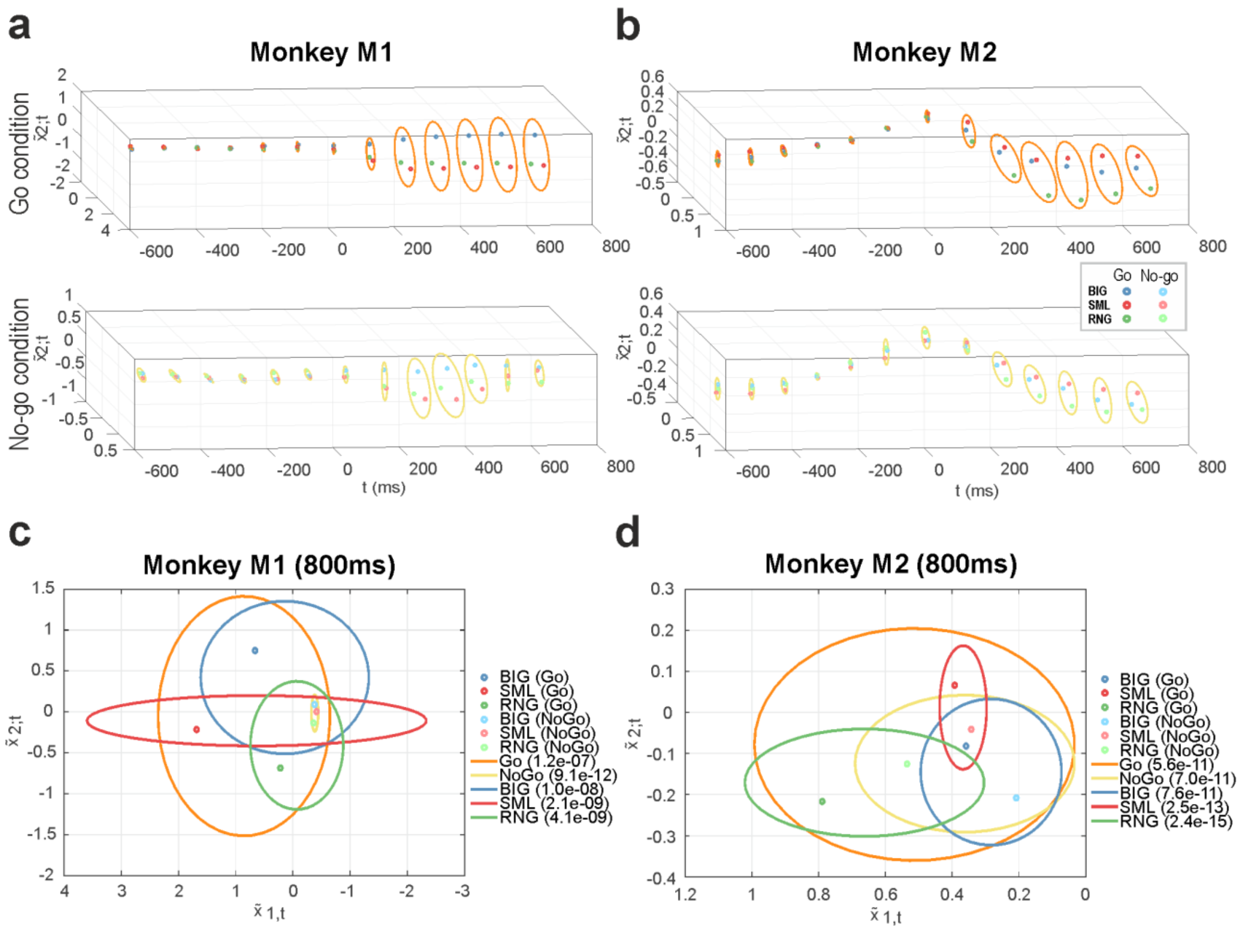

Figure S2. "Spread" of the single-trial neural trajectories for PT during Go and No-Go conditions. The spread is calculated as distance in the 2D neural space formed by the first 2 principal components of PCA. (a,b) Dynamics over time of the average neural trajectories of M1 and M2, respectively, in 2D neural space. Ellipsoids show 95% confidence intervals. (c,d) Clusters formed by the neural trajectories of M1 and M2, respectively, at time 800. The figures also quantify the "spread" of the clusters; see the Methods for an explanation.

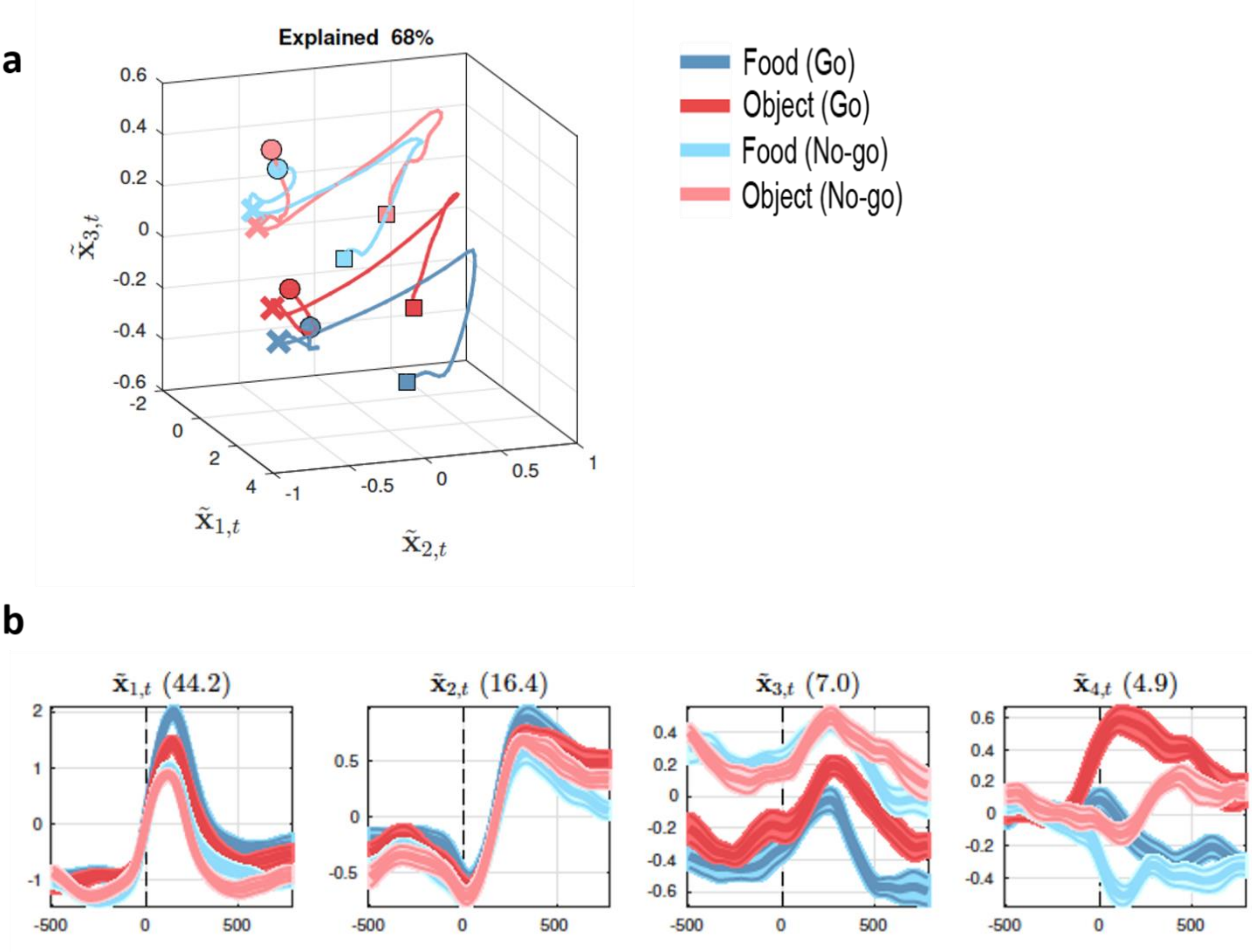

Figure S3. Neural space analysis with aggregated data from M3 and M4 - semantic task (ST). (a) Trajectories in the 3D "neural space" formed by the first three principal components of PCA, during the pragmatic task (PT). The neural trajectories span the interval [-500, 800], with zero being the target onset, and each bin representing the average of 200 ms. Circles mark the beginning of the neural trajectories, crosses mark target onset, and squares mark the end of the neural trajectories. (b) Trajectories in the 1D space of the first 4 components of PCA (one panel for each component). Mean (coloured line) and variance (coloured shades) are shown.

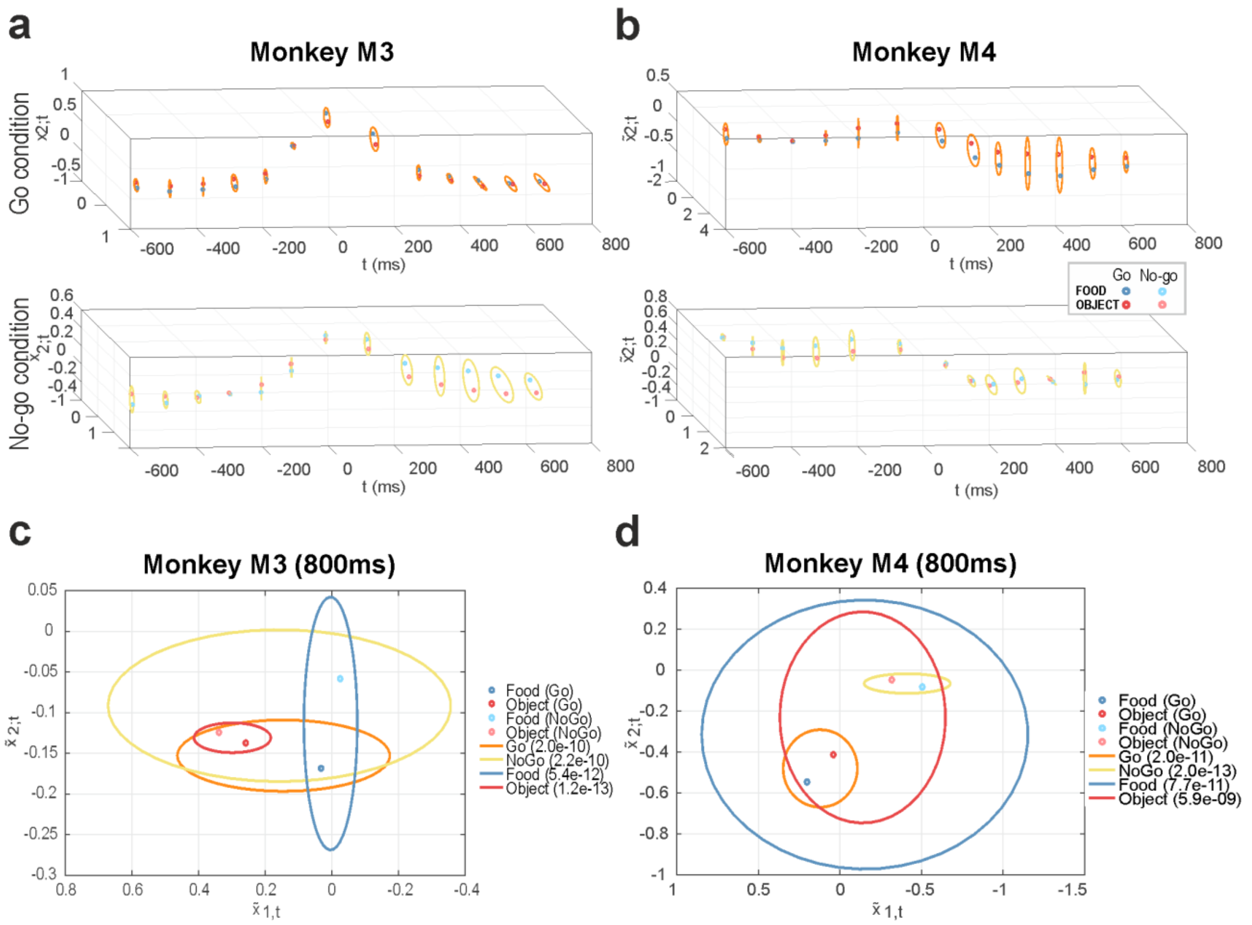

Figure S4. "Spread" of the single-trial neural trajectories for ST during Go and No-Go conditions. The spread is calculated as distance in the 2D neural space formed by the first 2 principal components of PCA. (a,b) Dynamics over time of the average neural trajectories of M3 and M4, respectively, in 2D neural space. Ellipsoids show 95% confidence intervals. (c,d) Clusters formed by the neural trajectories of M3 and M4, respectively, at time 800. The figures also quantify the "spread" of the clusters; see the Methods for an explanation.
